# A bedside test for methemoglobinemia, Sri Lanka

**DOI:** 10.2471/BLT.15.158147

**Published:** 2016-06-14

**Authors:** Fathima Shihana, Andrew H Dawson, Nicholas A Buckley

**Affiliations:** a South Asian Clinical Toxicology Research Collaboration, Faculty of Medicine, University of Peradeniya, Peradeniya, Sri Lanka.; b NSW Poisons Information Centre, Sydney Children’s Hospital Network, Australia.; c Sydney Medical School, University of Sydney, Sydney, Australia.

## Abstract

**Problem:**

Propanil is an aniline herbicide that is widely used for rice cultivation, but is also used for self-poisoning. Toxicity from propanil is largely due to methemoglobinemia. In resource-poor settings, the capacity to determine methemoglobin concentration is insufficient and prevents effective case management, which results in increased deaths from propanil poisoning.

**Approach:**

Blood with a methemoglobin concentration greater than 15% of total haemoglobin levels appears brownish in colour. We introduced a colour reference chart that can be used to semiquantitatively determine methemoglobinemia. Each ward in three rural hospitals received a chart. Ward staff, medical officers and trainee doctors were given a presentation describing the test method and how it should be used with the relevant national treatment guidelines.

**Local setting:**

In three rural hospitals in Sri Lanka, 401 patients were admitted with a diagnosis of propanil poisoning before the introduction of this test (2003–2007) and 262 patients after it was introduced (2008–2014), 46 of 663 patients died.

**Relevant changes:**

The chart can be freely produced with any good-quality colour printer. In three rural hospitals, deaths from propanil poisoning fell from 10% of those admitted with this diagnosis in 2003–2007 (38/401) to 3% (8/262) in 2008–2014 and the use of methylene blue increased from 10% (13/136) to 55% (59/107) over this period.

**Lessons learnt:**

This simple bedside test was associated with increased use of the first line treatment for propanil poisoning and improved survival. In 2011, the test was included in the national guidelines for the management of propanil poisoning.

## Résumé

**Problème:**

Le propanil est un herbicide à base d'aniline largement utilisé dans les cultures rizicoles, mais aussi dans les cas d'auto-empoisonnement. La toxicité du propanil se manifeste essentiellement par une méthémoglobinémie. Les établissements aux ressources limitées ne sont pas en mesure de déterminer la concentration de méthémoglobine, ce qui empêche la prise en charge efficace des patients et la réduction du nombre de décès par empoisonnement au propanil.

**Approche:**

Le sang dont la concentration en méthémoglobine est supérieure à 15% du taux d'hémoglobine total a une couleur brunâtre. Nous avons mis au point un nuancier de couleurs qui peut être utilisé pour déterminer semi-quantitativement le taux de méthémoglobine. Un nuancier de ce type a été remis à chacun des services de trois hôpitaux ruraux. Une description de la méthode de test et de la manière de l'utiliser dans le cadre des directives thérapeutiques nationales a été présentée au personnel de ces services, aux médecins ainsi qu'aux internes.

**Environnement local:**

Dans trois hôpitaux ruraux du Sri Lanka, 401 patients ont été admis avec un diagnostic d'empoisonnement au propanil avant l'introduction de ce test (2003–2007) et 262 patients après son introduction (2008–2014); 46 des 663 patients sont décédés.

**Changement significatifs:**

Le nuancier peut être produit librement avec n'importe quelle imprimante couleur de bonne qualité. Dans les trois hôpitaux ruraux, le nombre de décès par empoisonnement au propanil est passé de 10% des personnes admises avec ce diagnostic en 2003–2007 (38/401) à 3% (8/262) en 2008–2014, et l'utilisation de bleu de méthylène est passée de 10% (13/136) à 55% (59/107) sur cette période.

**Leçons tirées:**

Ce test simple au chevet du patient a été associé à une augmentation des traitements de première intention des empoisonnements au propanil ainsi qu'à une amélioration de la survie. En 2011, il a été inclus dans les directives nationales sur la prise en charge des empoisonnements au propanil.

## Resumen

**Problema:**

El propanil es un herbicida de anilina muy utilizado para el cultivo de arroz, pero también se utiliza para la autointoxicación. La toxicidad del propanil se debe principalmente a la metahemoglobinemia. En entornos de escasos recursos, la capacidad para determinar la concentración de metahemoglobina no es suficiente e impide el tratamiento eficaz de los casos, lo que resulta en un mayor número de muertes por intoxicación con propanil.

**Enfoque:**

La sangre con una concentración de metahemoglobina superior al 15% de los niveles totales de hemoglobina es de color amarronado. Se introdujo un gráfico de referencia de colores que puede utilizarse para determinar la metahemoglobinemia de forma semicuantitativa. Todas las plantas de tres hospitales rurales recibieron un gráfico. El personal de la planta, los oficiales médicos y los médicos en prácticas asistieron a una presentación que describía el método de la prueba y cómo ha de utilizarse con las directrices de tratamiento nacionales correspondientes.

**Marco regional:**

En tres hospitales rurales de Sri Lanka, 401 pacientes fueron admitidos con un diagnóstico de intoxicación con propanil antes de la introducción de esta prueba (2003–2007) y, después de su introducción, 262 (2008–2014). Murieron 46 de los 663 pacientes.

**Cambios importantes:**

El gráfico se puede obtener de forma gratuita con cualquier impresora a color de buena calidad. En tres hospitales rurales, las muertes por intoxicación con propanil cayeron de un 10% de los pacientes admitidos con este diagnóstico en 2003–2007 (38/401) a un 3% (8/262) en 2008–2014, y el uso de azul de metileno aumentó de un 10% (13/136) a un 55% (59/107) durante este periodo.

**Lecciones aprendidas:**

Esta sencilla prueba de cabecera se asoció con el aumento del uso del tratamiento de primera línea para la intoxicación con propanil y un mayor nivel de supervivencia. En 2011, se incluyó la prueba en las directrices nacionales para la gestión de la intoxicación con propanil.

## ملخص

**المشكلة:**

يعتبر البروبانيل أحد مبيدات الأعشاب المصنوعة من مركب الأنيلين والذي يتم استخدامه على نطاق واسع في زراعة الأرز، ولكن يمكن استخدامه أيضًا في التسميم الذاتي. ويعود السبب بشكل كبير في نسبة السُمية التي تنبعث من البروبانيل إلى مستوى الميتهيموغلوبين في الدم. إن مستوى القدرة على تحديد نسبة تركيز الميتهيموغلوبين في الدم غير كافٍ في المواقع التي تفتقر إلى الموارد، كما أنه يعيق الإدارة الفعالة للحالات مما يتسبب في حدوث وفيات بشكل متزايد نتيجةً للإصابة بتسمم من مادة البروبانيل.

**الأسلوب:**

إن الدم الذي تزداد فيه نسبة تركيز الميتهيموغلوبين عن 15% من إجمالي مستويات الميتهيموغلوبين يصبح لونه مائلاً للبني. لقد قمنا بإنشاء مخطط مرجعي لدرجات الألوان يمكن استخدامه لتحديد مستوى الميتهيموغلوبين في الدم بشكل تقريبي. وتم تسليم مخطط لكل جناح في ثلاث مستشفيات بالمناطق الريفية. كما تم إلقاء عرض تقديمي على طاقم العاملين بالأجنحة، ومسؤولي الخدمات الطبية، والأطباء المتدربين لتوضيح أسلوب الاختبار وكيفية استخدامه بما يتفق مع المبادئ التوجيهية القومية ذات الصلة بالعلاج.

**المواقع المحلية:**

في ثلاث مستشفيات بالمناطق الريفية في سري لانكا، تم إدخال 401 مريضاً بتشخيصٍ تضمن إصابتهم بتسمم نتيجة التعرض لمادة البروبانيل وذلك قبل تقديم هذا الاختبار (بين عامي 2003 و2007) وتم إدخال 262 مريضاً بنفس التشخيص بعد تقديم هذا الاختبار (بين عامي 2008 و2014)، وتعرض 46 مريضًا للوفاة من أصل 663 مريضاً.

**التغييرات ذات الصلة:**

يمكن بكل حرية إنشاء المخطط باستخدام أي طابعة ألوان تتميز بجودة مناسبة. في ثلاث مستشفيات بالمناطق الريفية، انخفضت نسب حالات الوفاة نتيجة الإصابة بتسمم من مادة البروبانيل من 10% من أصل عدد المرضى الذين تم إدخالهم إلى المستشفي بذلك التشخيص فيما بين عامي 2003 و2007 (38/401) إلى 3% (8/262) فيما بين عامي 2008 و2014 وازدادت نسب استخدام مركب الميثيلين الأزرق من 10% (13/136) إلى 55% (59/107) خلال هذه الفترة.

**الدروس المستفادة:**

إن ذلك الاختبار السريري البسيط مرتبط بالاستخدام المتزايد للأسلوب الأول المخصص لعلاج حالات التسمم من مادة البروبانيل وقد ساعد في التحسين من نسب النجاة. في عام 2011، تم إدراج هذا الاختبار ضمن المبادئ التوجيهية القومية لإدارة حالات التسمم من مادة البروبانيل.

## 摘要

**问题:**

敌稗是一种苯胺除草剂，被广泛用于水稻种植，但也同样被用于自体中毒。 敌稗的毒性主要源于高铁血红蛋白症。 在资源匮乏的环境中，对高铁血红蛋白浓度的确定能力不足，使得有效的病例管理无法实施，从而导致了敌稗中毒死亡数增加。

**方法:**

高铁血红蛋白含量超过整体血红蛋白数的 15% 时，血液呈褐色。 我们引入了一个可以通过半定量方法确定高铁血红蛋白症的颜色参照表。 我们为三个乡村医院的每个病房分发了一份该对照表。 并对病房医护人员、医疗官员以及进修医生做了一场描述该试验方法以及如何将其同相关国家性治疗指南一起使用的报告。

**当地环境:**

斯里兰卡的三个乡村医院中，有 401 位患者在该试验引入之前 (2003-2007) 因确诊为敌稗中毒而住院；有 262 位患者在引入之后 (2008–2014) 住院，663 位患者中，死亡 46 例。

**相关变化:**

该对照表可通过任何优质彩色打印机自由打印。 在三家乡村医院中，确诊入院患者中的敌稗中毒死亡数从 2003–2007 年间的 10% (38/401) 降至 2008–2014 年间的 3% (8/262)，并且在此期间，亚甲蓝的使用率从 10% (13/136) 增加至 55% (59/107)。

**经验教训:**

该项简单的病房试验与敌稗中毒一线治疗的增加以及生还率的提升有关。 2011 年，该项试验被纳入全国性敌稗中毒管理指南中。

## Резюме

**Проблема:**

Пропанил — это анилиновый гербицид, широко применяемый для выращивания риса, который также может быть использован для самоотравления. Токсическое действие пропанила по большей части связано с метгемоглобинемией. В условиях нехватки ресурсов отсутствует достаточный потенциал для определения концентрации метгемоглобина, поэтому осуществлять эффективное лечение невозможно. Это является причиной большого количества смертей вследствие отравления пропанилом.

**Подход:**

Кровь с концентрацией метгемоглобина, превышающей 15% от суммарного уровня гемоглобина, имеет коричневатый оттенок. Авторы представили справочную цветовую схему для полуколичественного определения метгемоглобинемии. Схема была предоставлена в каждое отделение трех сельских больниц. Для персонала, медицинских сотрудников и врачей-практикантов отделений провели презентацию, в которой описывался способ тестирования и порядок его применения с соблюдением соответствующих национальных руководящих принципов лечения.

**Местные условия:**

В трех сельских больницах Шри-Ланки 401 пациент поступил с диагнозом «отравление пропанилом» до того, как было введено данное тестирование (2003–2007 гг.), и 262 пациента поступили после его введения (2008–2014 гг.). Умерло 46 из 663 пациентов.

**Осуществленные перемены:**

Схема может быть самостоятельно изготовлена с помощью любого качественного цветного принтера. В трех сельских больницах количество смертей от отравления пропанилом снизилось с 10% от количества поступивших с таким диагнозом в 2003–2007 гг. (38 из 401) до 3% (8 из 262) в 2008–2014 гг., а количество случаев использования метиленового синего выросло с 10% (13 из 136) до 55% (59 из 107) за этот период.

**Выводы:**

Введение этого простого метода тестирования по месту оказания помощи обусловило более частое применение лечения первого ряда при отравлении пропанилом и большую выживаемость. В 2011 году метод тестирования был включен в национальные руководящие принципы ведения больных с отравлением пропанилом.

## Introduction

Propanil is an aniline herbicide that is widely used for rice cultivation. However, it is also used for deliberate self-poisoning. Propanil poisoning is usually manifested by methemoglobinemia and haemolytic anaemia, disorders that lead to reduction in effective oxygen transport and hypoxia.^1^ In general, the severity and symptoms of propanil poisoning correlate with the methemoglobin level in the blood. The recommended first-line treatment of methemoglobinemia is intravenous administration of methylene blue, which reduces the heme group from methemoglobin to hemoglobin. Methylene blue is inexpensive, accessible and easy to administer. The initial dose is 1–2 mg/kg of body weight with repeated doses titrated against the clinical response. Alternative treatments, such as ascorbic acid or N-acetylcysteine, are less effective, but can be used if methylene blue is unavailable. Exchange blood transfusion is only recommended when methylene blue or alternative treatments are ineffective.^2^

**Fig. 1:**
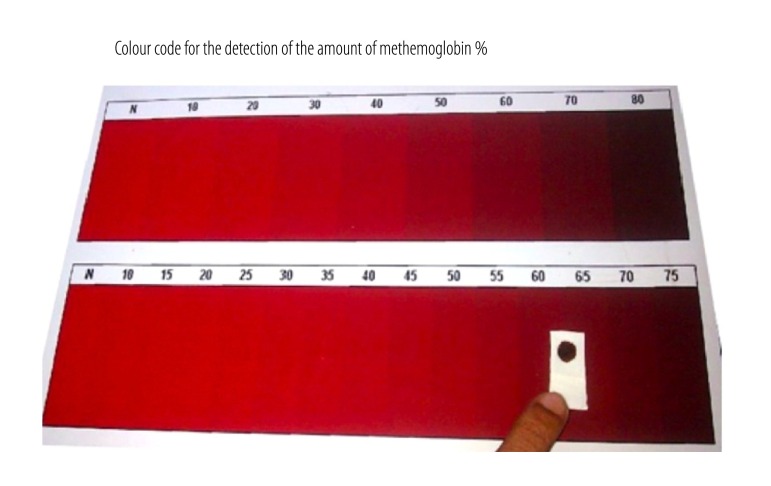
Semiquantitative estimation of methemoglobin levels in blood

The gold standard for determining methemoglobin concentration in the blood is by spectrophotometry. However, in rural areas, laboratory services are limited and it is usually not possible to measure methemoglobinemia with spectrophotometers or co-oximeters. As a methemoglobin concentration greater than 15% of total haemoglobin levels gives a brownish colour to blood, concentrations above 15% can be detected visually. Clinicians can use this visual method to diagnose patients, but to measure response to methylene blue treatment they need a more accurate method of quantification. We therefore developed a simple and low-cost bedside test for semiquantitative estimation of methemoglobin levels. The test is described in detail elsewhere.^3^ One drop of blood is placed on white absorbent paper and the colour of the blood spot compared to a reference chart (Fig. 1),^3^ corresponding to an estimated level of methemoglobinemia. We describe lessons learnt when using this bedside test in three hospitals in Sri Lanka.

(© Fathima Shihana)

## Local setting

In Sri Lanka, propanil poisoning has been recognized as a significant clinical problem. Two cohort studies – from two rural tertiary referral hospitals – have shown propanil self-poisoning case-fatality of 11% (45/412).^4^^,^^5^ In these cohort studies, we identified treatment patterns at odds with national guidelines for the treatment of methemoglobinemia published by the Sri Lankan National Poisons Centre. The guidelines recommended the use of methylene blue as a primary treatment.^2^^,^^6^ We observed relatively low use of methylene blue and higher use of ascorbic acid or exchange transfusion. Although clinicians reported that it was easy to diagnose propanil poisoning, the lack of methemoglobin concentration measures made it difficult to determine whether patients were responding to methylene blue. The lack of an objective measure against which to titrate the dose lead to a recurrence of methemoglobinemia in some patients.^4^^,^^5^ Clinicians also raised two additional concerns in using methylene blue and in managing the subsequent reductions in methemoglobin concentration. First, methylene blue is ineffective in patients with complete glucose-6-phosphate dehydrogenase (G6PD) deficiency and may cause hemolysis.^7^^,^^8^ About one in every 20 people from Sri Lanka has G6PD deficiency.^9^ Second, it is known that excessive doses of methylene blue can, in theory, cause or worsen methemoglobinemia.^3^

## Relevant changes

In collaboration with clinicians at three tertiary rural hospitals, we introduced the methemoglobin colour chart into all the medical wards in 2008. For ward staff, medical officers and trainee doctors, clinical research staff gave a single 20-minute oral presentation describing the test method,^3^ and how it could be used within the current national treatment guidelines. We provided each ward with a methemoglobin colour chart. The chart was prepared using a good quality printer in a local photo studio. The accuracy of the reproduction of the colour chart was checked with colour analysis that compared its colours to the original colour values derived in the test’s development.^3^ Each ward received a poster that described the use of the colour chart and reiterated the national treatment guidelines. After introducing the colour chart in the hospitals, we validated the accuracy of the test in the field by taking an additional sample from 13 patients and analysing the blood using a spectrophotometer.^3^

Independent of the treating team, clinical research assistants examined all poisoned patients until discharge or death. Clinical outcomes and treatment of each patient was prospectively recorded into the cohort database. We then identified, from the database, data on patients admitted with propanil poisoning. We were able to examine outcomes in 401 patients before (2003–2007) and 262 patients after (2008–2014) the introduction of the test.^10^

## Lessons learnt

After we introduced the test, case-fatality for propanil poisoning fell by two-thirds, from 10% (38/401) to 3% (8/262). Retrospective examination of available patient medical records showed an increase in the use of methylene blue after the test was introduced: from 10% (13/136) to 55% (59/107).^10^ Records showed that titrated doses of methylene blue were more common than single-dose treatment and such dosage patterns have been sustained. The use of less effective treatments and exchange transfusion were reduced,^10^ suggesting that clinicians accepted the utility of this test in the management of methemoglobinemia.^10^

Our report has limitations. It is possible that some of the survivals may have been from unrelated improvements in care at this time. Our quasi-experimental comparison of two different time periods cannot exclude alternative explanations for the changes in number of deaths. However, the change in management and reduction in deaths was considerable and no major change in these outcomes had been observed within the study locations in the six years before the intervention.

Box 1 summarizes the main lessons learnt. The uptake of the test into practice was high, as the test quickly provided results, allowing more informed use of national treatment guidelines. Distribution of the colour chart was cheap, as it could be printed from a freely available file by any good-quality colour printer.^11^

Box 1 **Summary of main lessons learnt**

- Low-cost bedside estimation of methemoglobin levels changed case management and was associated with a reduction in deaths from propanil poisoning.
- The test helped doctors to provide treatment consistent with national guidelines for management of poisoning.
- Applied research that addresses local clinical concerns can be translated into practice and better health outcomes.

The simplicity and low cost of the test presented here can facilitate the treatment of methemoglobinemia in resource-poor settings. The test was included in the 2011 edition of the national guidelines: *Management of poisoning*^6^ and in the curriculum of local postgraduate training programmes for clinicians and nurses.
